# Current and emerging approaches to manage chronic inflammatory gut disorders

**DOI:** 10.3389/fcimb.2026.1762119

**Published:** 2026-03-04

**Authors:** Katelyn M. Green, Sang-Min Shin, Ramesh K. Jha, Anand Kumar

**Affiliations:** 1 Microbial and Biome Sciences Group (B-IOME), Bioscience Division, Los Alamos National Laboratory, Los Alamos, NM, United States; 2 Nuclear & Radiochemistry Group (C-NR), Chemistry Division, Los Alamos National Laboratory, Los Alamos, NM, United States

**Keywords:** chronic inflammatory gut disorders, diagnosis and treatment, living whole cell sensor, nanomedicine, probiotics, synthetic circuit engineering

## Abstract

Chronic inflammatory gastrointestinal disorders, including inflammatory bowel disease (IBD), Crohn’s disease, ulcerative colitis, and irritable bowel syndrome (IBS), remain challenging to manage due to complex etiologies, heterogeneous disease progression, and limitations in current diagnostic and therapeutic strategies. Existing clinical approaches rely on a combination of invasive and non-invasive diagnostic tools, while therapeutic management predominantly involves symptomatic control, disease-modifying pharmacotherapy, and surgical interventions. However, these strategies often fail to enable early or real-time disease detection and frequently fall short of achieving sustained remission. This review highlights two emerging and potentially transformative approaches: nanomedicine and living diagnostic–therapeutic systems. Nanomedicine has gained significant attention for its ability to enhance targeted drug delivery and improve therapeutic efficiency, addressing several limitations of conventional treatments; nevertheless, challenges related to delivery consistency, biosafety, scalability, and long-term efficacy persist. In parallel, living diagnostic–therapeutic systems—engineered whole-cell sensors capable of real-time sensing and on-demand therapeutic response within the gut—represent a compelling alternative. Although still at an early stage of development, promising preclinical and limited clinical studies demonstrate their potential utility. Key challenges remain, including biosensor functionality, genetic stability, microbial colonization, host–microbe interactions, and integration into existing healthcare frameworks, alongside regulatory and translational barriers. Overall, the convergence of nanomedicine and living, responsive systems may offer a transformative pathway for the diagnosis and treatment of chronic inflammatory gut diseases.

## 1 Introduction

Chronic inflammatory conditions affect approximately 51.8% of adults in the United States, with 27.2% of individuals experiencing multiple chronic conditions (Boersma et al., 2020). These range of conditions include inflammatory bowel disease (IBD), heart disease, diabetes, chronic kidney disease, non-alcoholic fatty liver disease, autoimmune disorders, degenerative disorders, and even cancer (Furman et al., 2019; Roth, 2018). Collectively, chronic inflammatory diseases account for over 50% of deaths worldwide (Furman et al., 2019; Roth, 2018).

IBD is a chronic, relapsing inflammatory disorder of the gastrointestinal tract and represents a major form of chronic intestinal inflammation, with a rapidly evolving global epidemiology (Wang et al., 2023; Yang et al., 2025). It primarily manifests as ulcerative colitis (UC) or Crohn’s disease (CD), distinguished by differences in disease location, histopathology, and clinical presentation. Recent comprehensive reviews have discussed these conditions in detail, including their pathogenesis, diagnosis, and therapeutic management (Appiah et al., 2025; Eltantawy et al., 2023; Seyedian et al., 2019). Here, we summarize the key distinctions between UC and CD in **Table 1** to provide a concise snapshot of their defining features, drawing on these published studies.

**Table 1:** Key anatomical, histological, and clinical differences between ulcerative colitis and Crohn’s disease.

Features	CD	UC
Anatomical distribution		
GI tract involvement	Mouth to anus	Colon only
Most common regions	Terminal ileum, ileocecal	Colon and rectum
Lesion pattern	Discontinues	Continuous
Small intestine involvement	Yes, most common	No
Rectal involvement	Maybe	Always present
Symptomatic difference		
Diarrhea	Chronic, often non bloody	Bloody diarrhea common
Abdominal pain	Frequent, lower right abdomen	Mild to moderate, lower right abdomen
Rectal bleeding and tenesmus	Rare	Common
Weight loss and malabsorption	Common	Rare
Fever	Common during active disease	Rare
Histopathological Differences		
Depth of inflammation	Transmural	Limited to mucosa and submucosa
Granulomas	Maybe present	Absent
Crypt abscesses	May occur	Common
Ulceration	Deep liner ulcers	Superficial diffused ulcers
Fibrosis	Common	Rare
Fistula	Common	Absent
Disease Severity and Complications		
Overall severity	Often more severe and progressive	Variable (mild to severe)
Major complications	Strictures, fistulas, abscesses	Toxic megacolon, severe hemorrhage
Colorectal cancer risk	Increased	Higher than CD
Surgical outcome	Disease may recur after resection	Colectomy is curative
Nutritional deficiencies	Common	Rare

**Figure 1:**
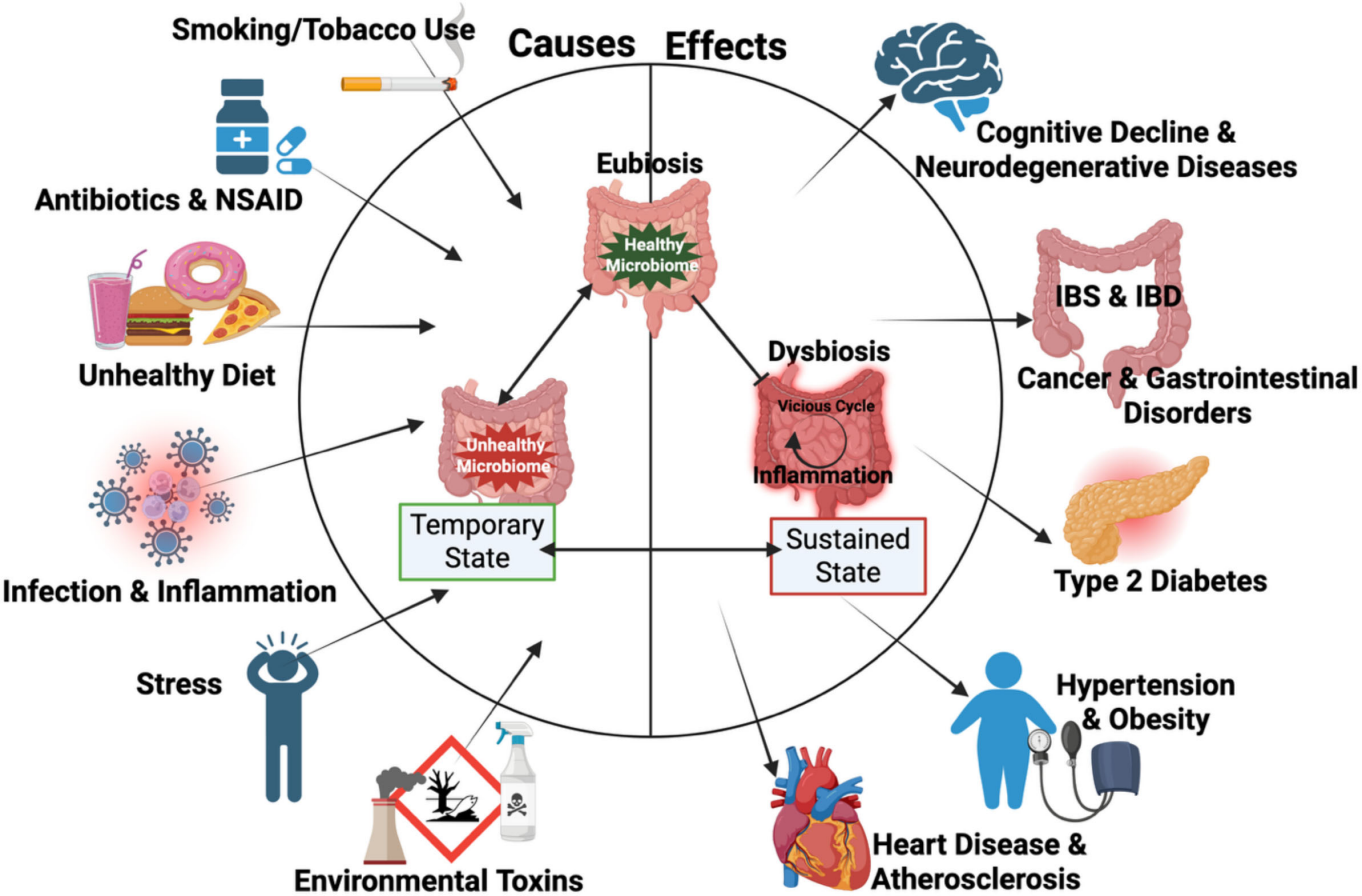
Causative factors of dysbiosis, an imbalance in the gut microbiome, can disrupt the ratio of harmful to beneficial bacteria, leading to inflammation. Sustained inflammation is linked to adverse health outcomes. Figure created with BioRender.

Recent studies increasingly associate perturbed gut microbiota referred to as dysbiosis as a potential root cause of chronic inflammatory conditions. This imbalance often originates in the gut and then influences systemic inflammation, promoting diseases beyond the gastrointestinal tract (Buttó and Haller, 2016) (**Figure 1**). When inflammation persists over a prolonged period, it often leads to severe and life-threatening health conditions.

Several factors have been identified as triggers of gut dysbiosis including poor diet, obesity, disrupted sleep, chronic infections, stress, lack of physical activity, exposure to pollutants or chemicals, frequent antibiotic use, use of nonsteroidal anti-inflammatory drugs (NSAID), and tobacco use (Furman et al., 2019) (**Figure 1**). These triggers can disrupt gut microbial balance, initiate inflammation in the gut, and eventually contribute to systemic inflammatory conditions. Inflammation is the immune response to injury, toxins, or infections (Ansar and Ghosh, 2016) and involves the activation of inflammatory cells and signaling pathways to eliminate external or internal insults (Chen et al., 2018). A key component of this process is the generation of reactive oxygen species (ROS), which plays a critical role in inflammatory diseases, including those of the gastrointestinal (GI) tract.

When the gut’s mucosal barrier and environment is compromised by inflammatory triggers, the innate immune system responds rapidly, initiating an acute inflammatory response in the lamina propria. Polymorphonuclear leukocytes (PMNs) are recruited to the site of infection or damage, where these cells engulf pathogens and release ROS, enzymes, and vasoactive and pro-inflammatory substances (Mittal et al., 2014). While ROS generation can help resolve inflammation by creating a hypoxic environment through oxygen depletion to activate anti-inflammatory pathways (Campbell et al., 2014), excessive and prolonged oxidative stress marked by an imbalance between ROS production and antioxidant defense can result in apoptosis and tissue damage (Rezaie et al., 2007). The chronic inflammatory state arises when immune cells and pathways remain persistently activated even in the absence of external threats. If left unchecked for months or years, this chronic inflammation can impair organ and tissue function (Oronsky et al., 2022). Therefore, the elevated levels of pro-inflammatory markers and ROS are linked to the development of numerous diseases, including cancer, atherosclerosis, diabetes, arthritis, and cognitive decline (Furman et al., 2019; Ranneh et al., 2017). Given the profound health consequences associated with chronic gut inflammation, early diagnosis, management, and treatment are essential for maintaining overall health and preventing long-term complications.

In this review, we briefly outline current diagnostic and therapeutic approaches for chronic inflammatory gut conditions and discuss their limitations. We then describe recent advances aimed at addressing these challenges, with particular emphasis on emerging nanomedicine-based and smart, responsive therapeutic strategies. Promising studies that integrate diagnostic and therapeutic functionalities are highlighted, underscoring their potential to improve disease monitoring and enable personalized treatment. We also discuss the key barriers to translating these emerging approaches into clinical application in humans.

## 2 Current approaches to diagnose and treat chronic inflammatory gut conditions

### 2.1 Diagnosis

**Figure 2:**
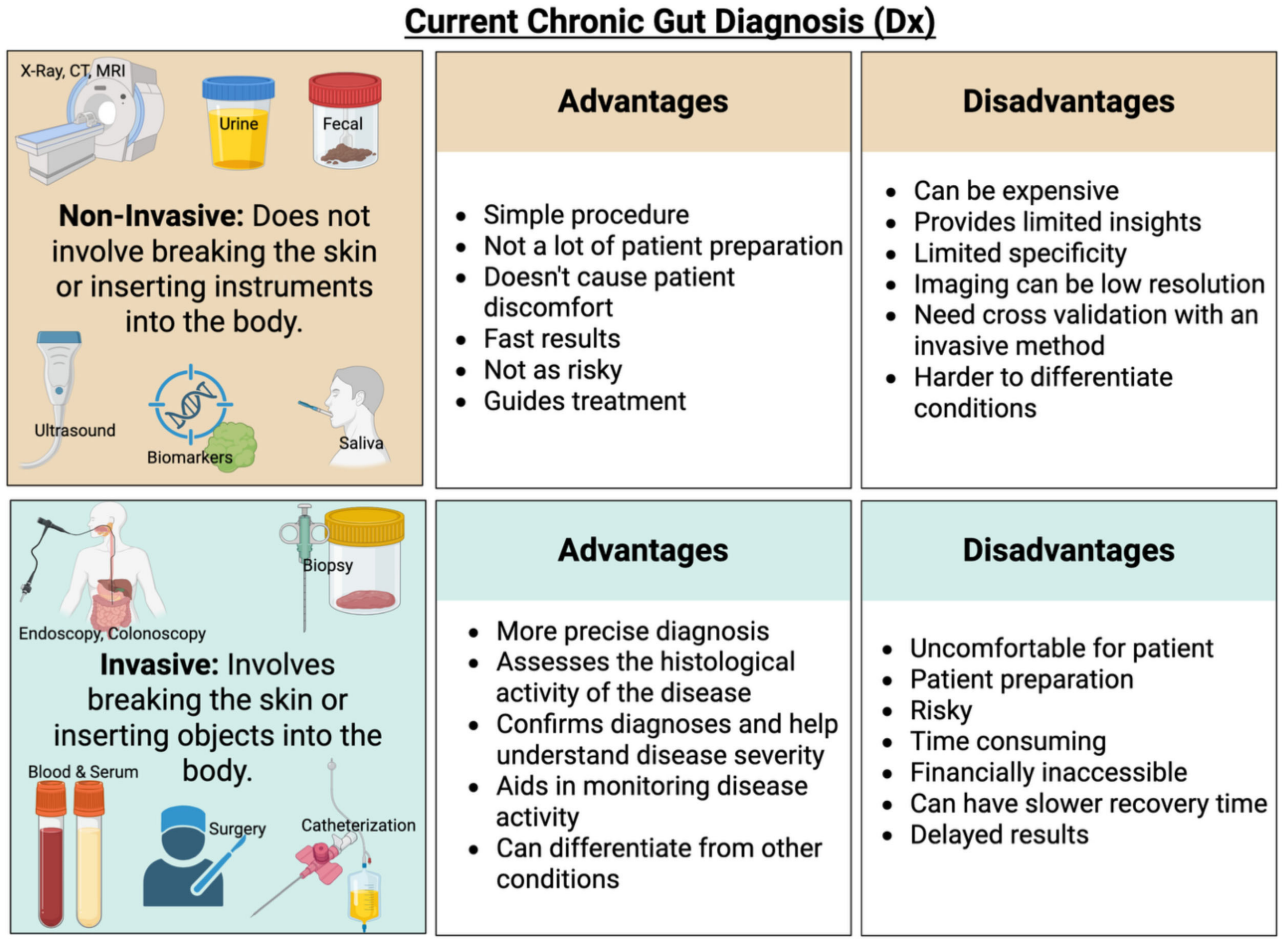
An overview of current diagnostic methods for gut inflammatory conditions, encompassing both non-invasive and invasive approaches. Each method carries its own advantages and limitations. The figure was created using BioRender.

Diagnosing chronic gut inflammatory conditions is challenging due to the general nature of GI symptoms, which can vary significantly between individuals and often lack hallmark indicator symptoms. As a result, many individuals are not diagnosed until the condition progresses to a more serious or even life-threatening stage. Current diagnostic methods for managing chronic inflammatory diseases have varying degrees of limitations, as described (Tontini et al., 2015) (**Figure 2****).** Broadly, diagnostic approaches for chronic inflammatory conditions can be categorized into two types: invasive and non-invasive.

As the names suggest, *invasive approaches* include biopsies, where tissue samples are collected from parts of the GI tract for analysis. A semi-invasive method is endoscopy, which allows direct visualization of the GI tract to identify pathophysiological changes (Shodeinde et al., 2020). Gastrointestinal endoscopy is often performed in conjunction with biopsy collection (Shodeinde et al., 2020). For example, when diagnosing IBD, an ileocolonoscopy is commonly recommended, during which multiple biopsy samples are taken from different regions of the intestine (Peixoto et al., 2016). Depending on the location of symptoms and the patient’s medical history, upper or lower GI endoscopies and biopsies are performed to examine the affected areas of the intestine (Peixoto et al., 2016). Video capsule endoscopy and device-assisted enteroscopy are emerging techniques used to examine the intestine and aid in the diagnosis of IBD by evaluating the disease’s histological activity. These tools are essential for assessing remission, guiding treatment decisions, and providing a comprehensive analysis of disease progression (Hong and Baek, 2024). Another invasive diagnostic approach involves collecting bodily fluids, such as blood or serum, to detect biomarkers associated with inflammation. In terms of invasiveness and patient discomfort, the diagnostic procedures can be ranked (from least to most invasive) as follows: bodily fluid collection, endoscopy, and biopsy. More invasive approaches often carry health risks, are labor-intensive, require patient preparation, cause discomfort, and may be financially inaccessible—particularly in countries like the United States, where healthcare can be prohibitively expensive (Tontini et al., 2015).

*Non-invasive approaches* include the detection of biological markers such as enzymes, antibodies, or specific proteins (e.g., lactoferrin, calprotectin, and C-reactive protein in bodily fluids that can be collected non-invasively, such as urine, fecal matter, or saliva (Hickman, 2024; Lewis, 2011). However, biomarker detection in these fluids often does not provide a complete or fully accurate picture of disease status. As a result, such findings typically require confirmation through invasive diagnostic methods (Tontini et al., 2015; Hong and Baek, 2024).

Identifying specific biomarkers for chronic inflammation remains challenging, as inflammation is a common feature across many diseases. Most biomarkers reflect generalized inflammation and cannot reliably distinguish between acute and chronic conditions, particularly in the gut (Furman et al., 2019; Iskandar and Ciorba, 2012). In clinical practice, biomarkers are used in conjunction with routine testing to support but not independently confirm. They can help differentiate Crohn’s disease from ulcerative colitis, assess disease activity, stratify patients, and predict therapeutic response (Iskandar and Ciorba, 2012). For certain gastrointestinal conditions like gastric cancer, diagnosis still relies heavily on invasive procedures, particularly in early stages. In this context, ongoing research is focused on developing non-invasive diagnostic techniques, such as genetic and metabolic biomarkers, to improve early detection and screening (Orăşeanu et al., 2023).

Body imaging techniques, including X-ray, MRI, and CT scans, are also considered non-invasive methods for diagnosing chronic inflammatory gut conditions. These techniques allow direct visualization of structural abnormalities in the GI tract, unlike biomarkers, which provide indirect evidence of disease. However, the accuracy and utility of imaging can depend on the method used and may require patient preparation, such as the administration of contrast agents (especially for X-rays and CT scans). While imaging methods can be very informative, they come with limitations: radiation exposure, high costs, limited availability, and they are not always recommended as first-line diagnostic tools.

Another important non-invasive method is the use of clinical indices or scoring systems to assess chronic GI conditions, especially ulcerative colitis and Crohn’s disease. These indices evaluate disease severity based on various patient data, such as stool frequency, rectal bleeding, endoscopic findings, and the physician’s overall assessment (Iskandar and Ciorba, 2012). While these scoring systems are valuable, they often rely on data from invasive procedures, particularly endoscopy and biopsy, to be fully accurate and useful.

In conclusion, although non-invasive approaches are useful for initial screening and disease monitoring, they typically require validation through invasive diagnostic methods to confirm a diagnosis and guide treatment (Iskandar and Ciorba, 2012; Tontini et al., 2015).

#### 2.1.1 Limitations of current diagnosis methods

A major limitation of existing methods (both invasive and non-invasive) for diagnosing chronic inflammatory gut conditions is that they provide only a snapshot of disease status, rather than offering real-time assessment. These approaches are often not ideal for early detection, and invasive methods are not always a preferred first choice. Additionally, non-invasive biomarker detection is frequently non-specific, with false positives and false negatives being common (Watson et al., 2019). False positives can result in incorrect diagnoses, leading to unnecessary treatments with potential side effects, along with additional follow-up appointments, tests, or referrals. Conversely, false negatives may delay diagnosis, allowing the underlying condition to worsen (Watson et al., 2019). There is a clear need for next-generation, non-invasive diagnostic methods that can monitor gut inflammation in real-time and provide quantitative, accurate assessments. One promising innovation is the development of a living whole-cell biosensor, which could revolutionize the way chronic inflammatory gut conditions are detected and managed.

### 2.2 Treatment

**Figure 3:**
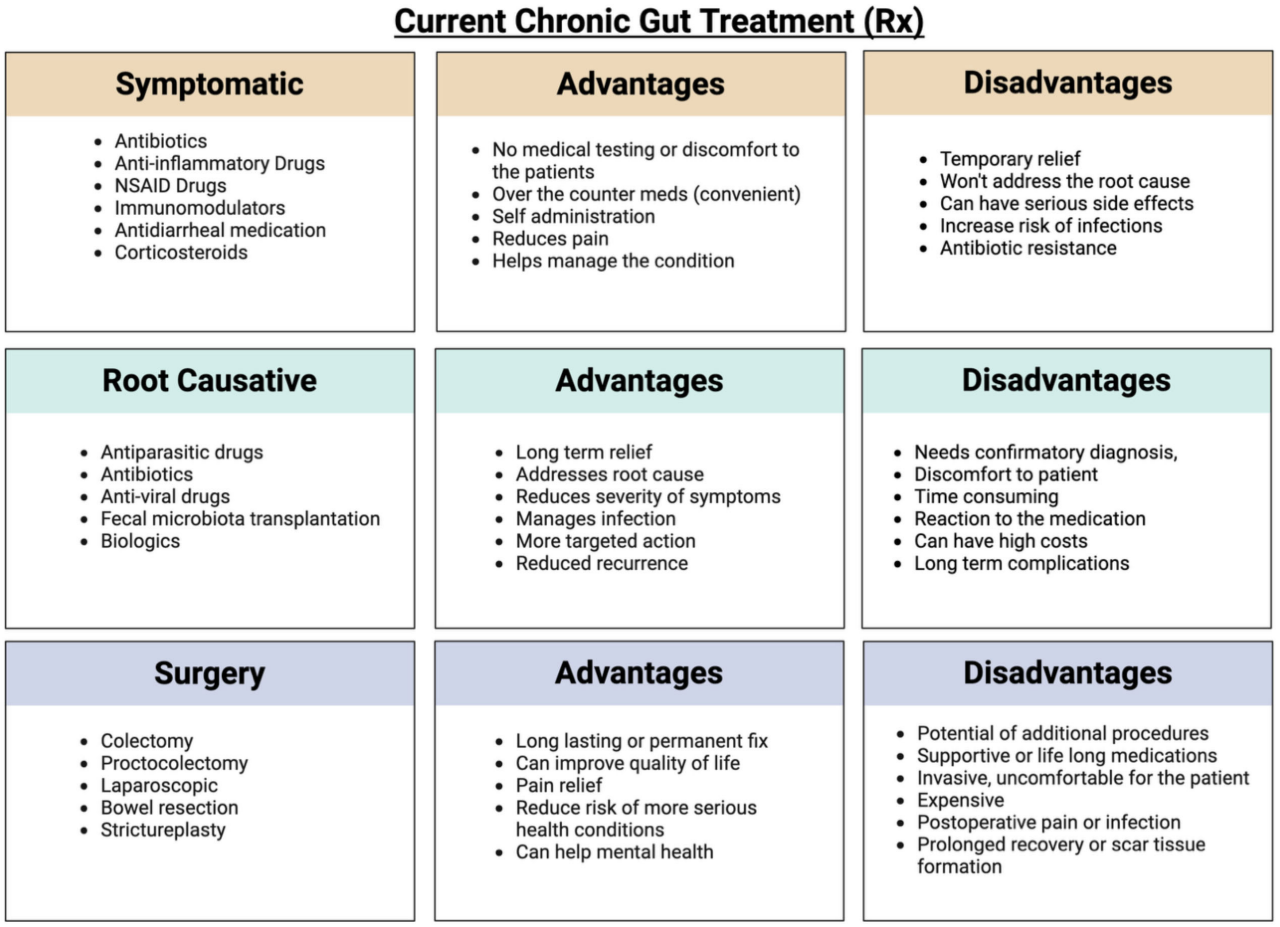
Current treatment methods for gut inflammatory conditions, categorized into symptomatic management, root-cause–targeting therapies, and surgical interventions. Each category offers distinct advantages and disadvantages. The figure was created using BioRender.

Treatment for managing chronic inflammatory gut conditions can be broadly categorized into three main approaches: symptomatic treatment, root-cause treatment, and surgical intervention as a last resort (**Figure 3**).

#### 2.2.1 Symptomatic treatment

Symptomatic treatment focuses on short-term symptom relief and typically involves the administration of anti-inflammatory drugs, antibiotics, steroids, immunosuppressants, and NSAIDs. These medications are intended to suppress symptoms temporarily. In addition, vitamins and supplements are often prescribed to support the healing process (Diaz-Borjon et al., 2009; Wongrakpanich et al., 2018). Standard treatment for IBD includes drugs such as mesalamine, azathioprine, glucocorticoids, and TNF agents (Roy and Dhaneshwar, 2023). These treatments aim to regulate the expression of TNF-α, a key mediator in the production of proinflammatory cytokines that drive IBD pathology (Sandborn, 2013; Sands and Kaplan, 2007). Despite this broad treatment approach, a significant number of IBD patients either do not respond initially or lose therapeutic response over time (Roy and Dhaneshwar, 2023). Treatment for Crohn’s disease is usually personalized based on individual risk factors, clinical considerations, and patient preferences. Initial therapy typically involves corticosteroids for symptom control, followed by anti-TNFα therapy with surgical intervention in more advanced or treatment-resistant cases. Nevertheless, more than half of Crohn’s disease patients eventually require surgery due to disease progression (Cushing and Higgins, 2021).

Symptomatic treatments generally do not address the root cause of the disease and may come with a range of side effects and health risks. These include an increased risk of heart attacks, strokes, kidney impairment, high blood pressure, and gastrointestinal bleeding (Davis and Robson, 2016). Moreover, these treatments are known to cause and sustain dysbiosis in the gut microbiome.

#### 2.2.2 Root cause treatment

Root-cause treatment is typically initiated only after a confirmatory diagnosis has been made and is most applied in cases of intestinal infections caused by bacteria, viruses, fungi, or parasites, with the appropriate medications selected based on the causative agent. Bacterial infections of the gut are usually treated with antibiotics, often in combination with medications that relieve symptoms (Ranasinghe and Fhogartaigh, 2021). Fungal and parasitic infections are managed using antifungal and antiparasitic drugs, respectively, also supported by symptom-relief medications (Cong et al., 2023). For viral gastrointestinal infections, treatment is usually symptomatic, as these infections tend to be self-limiting. Only in severe or complicated cases are antiviral drugs, including monoclonal antibodies, considered (Santos-Ferreira et al., 2021; Stuempfig et al., 2025). The key advantages of root-cause treatment are that it: targets the specific causative agent, is often of shorter duration, and relieves the condition with a lower risk of side effects compared to long-term symptomatic therapies. However, the growing problem of antimicrobial resistance can complicate treatment effectiveness (Tamma et al., 2024). Moreover, achieving a clear and early confirmatory diagnosis can be challenging, limiting the timely use of this ideal treatment approach.

#### 2.2.3 Surgery

Surgery becomes the last resort when a patient does not respond to symptomatic treatment, and root-cause treatment is either ineffective or not feasible due to the complexity of the chronic inflammatory condition (Ahmed Ali and Kiran, 2022; Chandrasinghe, 2022; Triantafillidis, 2024). It is typically considered when the disease progresses to severe complications that threaten the patient’s health or life. In such cases, the affected section of the intestine is surgically removed. The long-term impact of the surgery depends on the location and extent of the resection. Patients may require lifelong medication and adherence to a specialized diet following surgery (Irani et al., 2023; Nightingale and Woodward, 2006). While surgery can permanently resolve the immediate issue, it often results in a compromised lifestyle, including ongoing medical management and dietary restrictions (Irani et al., 2023). These situations usually arise from delayed diagnosis, when the condition has already progressed to an advanced or irreversible stage.

## 3 Nanomedicine: A promising approach to manage chronic inflammatory gut conditions

Nanomedicine utilizes nanoscale (10^−9^ m) molecules and structures to deliver therapies for diagnosis, and treatment of various disease conditions (Bayda et al., 2020; Huang et al., 2024; Mansoori and Soelaiman, 2005). Various studies have explored the application of nanotechnology in medicine. For example, nanoparticles (NPs) have been used as vaccine carriers and adjuvants, enhancing immunogenicity, achieving targeted delivery, improving bioavailability, and directly combating pathogens and inflammation (Azócar et al., 2019; Huang et al., 2024).

Various types of NPs have been explored for the diagnosis and treatment of IBD due to their ability to provide a stable and protective environment for encapsulated agents, shielding them from enzymatic degradation and the acidic conditions of the stomach (Date et al., 2016; Lundquist and Artursson, 2016; Zhang and Merlin, 2018; Stojanov and Berlec, 2024; Zhao et al., 2025). This property is particularly advantageous for targeted drug delivery to the inflamed intestine, where it can enhance therapeutic efficacy and reduce dosing frequency. Based on their composition and source, NPs can be broadly classified into lipid-based bilayer NP, polymeric NP (e.g., PLGA, chitosan), hydrogel-based NP, exosome-derived NP, and metallic NP (Stojanov and Berlec, 2024; Zhao et al., 2025). Each nanoparticle class exhibits distinct advantages and limitations, briefly summarized in **Table 2** and discussed in detail previous reviews (Yue et al., 2023; Chen and Li, 2025; Fei et al., 2022; Laroui et al., 2013; Jiang et al., 2024).

**Table 2:** Advantages and limitations of NP type used for diagnosis and treatment of inflammatory gut disorders (Alshawwa et al., 2022).

NP type	Examples	Advantages	Limitations
Lipid-based bilayer	Liposomes, solid lipid	High biocompatibility, encapsulation of both hydrophilic and hydrophobic drugs, enhanced cellular uptake, and clinically validated platforms	Limited stability, potential drug leakage, and rapid clearance by the reticuloendothelial system
Polymeric	PLGA, chitosan	Tunable size and degradation, controlled and sustained drug release, good mechanical stability, and scalable synthesis	Acidic degradation byproducts, batch variability, and potential polymer-dependent cytotoxicity
Hydrogel	PEG, alginate hydrogels	High water content, excellent biocompatibility, stimuli-responsive drug release (pH, enzymes, ROS), and suitable for inflamed tissue targeting	Lower mechanical strength, complex synthesis, and risk of premature swelling or drug release
Exosome-derived	Cell-derived exosomes	Natural origin, low immunogenicity, intrinsic targeting ability and efficient cellular uptake	Low yield, challenging isolation and scale-up, and limited standardization and drug-loading efficiency

In diagnostic imaging, dextran-coated cerium oxide NPs have been reported as a novel CT contrast agent for GI track imaging in patients with and without IBD. These NPs not only enhance imaging but also protect intestinal cells from oxidative damage, offering greater stability, biocompatibility, and target specificity toward inflammation sites compared to conventional CT contrast agents like iodinated small molecules or barium sulfates (Naha et al., 2020). Additionally, the co-administration of silymarin and selenium NPs in a rat model of colitis showed a promising antioxidant effect, reducing NF-κB-mediated proinflammatory cytokine production (Miroliaee et al., 2011). Another study engineered NPs to deliver the anti-inflammatory tripeptide (Lys-Pro-Val) directly to the colon in a murine colitis model. These NPs successfully released near the colonocytes, protecting against inflammatory responses (Laroui et al., 2010). Such studies demonstrate that NPs can serve as multifunctional drug delivery systems, modifiable in various ways to enhance treatment for IBD and other chronic inflammatory conditions.

Ongoing research is investigating the use of nanosensors and nanoprobes for detection, monitoring, and imaging of inflammatory lesions (Liu et al., 2024). However, concerns regarding the toxicity and bioaccumulation of inorganic NPs (such as gold, silver, and iron oxide) have prompted researchers to explore organic nanomaterials, including polymers, dendrimers, and liposomes, for safer therapeutic and diagnostic applications (He et al., 2023). While organic NPs are ideal for drug delivery because of their biocompatibility, they lack the tunability that metallic inorganic nanoparticles offer. Therefore, combining organic and inorganic nanoparticles into hybrid systems enhances versatility, stability, and multifunctionality (Pietersz et al., 2017). However, it is important to note that nontargeted NPs have rarely demonstrated convincing advantages that translate into clinical applications (Pietersz et al., 2017). Despite the surge in NPs research over the past decade, few NPs-based products have become commercially available.

Among metals, gold (Au) NPs are the most frequently used in biomedical applications due to their stability and low reactivity, although other metals like silver (Ag) and copper (Cu) have shown to possess promising properties (Shodeinde et al., 2020). A study found that Ag-NPs exhibit better sensing behavior than Cu-NPs across the entire infrared spectrum of the electromagnetic rays (Singh Sekhon and Verma, 2011).

Furthermore, semiconductor quantum dots (QDs) have been explored for biomedical use because of their unique optical properties. Their size, shape, and composition can be manipulated to achieve fluorescence at different wavelengths (Chan et al., 2002). Due to their much higher brightness compared to conventional organic dyes, there is significant interest in using NPs and quantum dots for biomedical imaging and biosensing. Moreover, they can be tagged to specific molecules, allowing for fluorescent detection of biomarkers (Knipe et al., 2013; Pinaud et al., 2006). Localized surface plasmon resonance (LSPR) biosensors are a promising class of sensors that confine light within metal nanostructures and measure changes in the local refractive index caused by the adsorption of target molecules (Brolo, 2012). These LSPR nano-biosensors are highly sensitive and can be easily integrated into miniaturized devices, making them ideal for point-of-care testing applications (Wang et al., 2017).

In a recent study, the authors exploited lipid-based NPs to evaluate the efficacy of encapsulated nucleic acids designed to modulate TNF-α splicing in both *in vitro* cell systems and *in vivo* murine models of colitis (Qassem et al., 2025). The encapsulated formulations were shown to be stable and well tolerated. Notably, lipid NPs preferentially accumulated in inflamed intestinal tissue at sufficient concentrations to elicit a therapeutic effect. However, the authors also observed biodistribution of these lipid NPs in other organs, suggesting the potential for off-target accumulation (Qassem et al., 2025). This finding underscores the need for improved targeting strategies to enhance tissue specificity and minimize systemic exposure. In a similar approach, IL-22 mRNA, a known for its potent anti-inflammatory and epithelial regenerative effects in ulcerative colitis was encapsulated in lipid NP and evaluated following oral administration in a murine UC model (Sung et al., 2022). Mice treated with IL-22 mRNA–loaded lipid NPs exhibited accelerated mucosal healing, as evidenced by improved body weight recovery, increased colon length, reduced histological injury scores, and decreased expression of pro-inflammatory cytokines. Collectively, these studies demonstrate that lipid-based NP serve as effective carriers for nucleic acid delivery, offering stability, safety, and therapeutic efficacy. With appropriate targeting modifications, lipid NPs hold significant potential for delivering a wide range of therapeutic cargos to specific tissues or organs in inflammatory bowel disease.

PLGA NPs have been used to encapsulate well-established anti-inflammatory therapeutics, including aminosalicylic acid and the monoclonal antibody adalimumab, both of which are commonly used in the management of IBD (Davoudi et al., 2018; Feng et al., 2025). These formulations were evaluated *in vitro* using cell culture and organoid systems. The studies demonstrated efficient cellular uptake of PLGA NPs and effective delivery of the encapsulated agents to inflamed cells, highlighting the potential of PLGA-based carriers for targeted drug delivery under inflammatory conditions. A similar strategy was applied in a murine colitis model, in which the Janus kinase inhibitor tofacitinib citrate—an FDA-approved therapy for ulcerative colitis—was delivered via PLGA NP encapsulation (Seegobin et al., 2025). The findings showed that approximately 80% of the drug was released in the colonic environment, and that degradation of PLGA by the colonic microbiota did not significantly alter drug release kinetics. Collectively, these results underscore the promise of PLGA NP-based delivery systems in enhancing therapeutic efficacy, enabling dose reduction, and potentially minimizing systemic side effects in IBD treatment.

Exosome-derived nanoparticles represent an emerging and promising platform for therapeutic delivery in IBD, owing to their low immunogenicity and superior tissue-targeting capabilities compared with many synthetic nanoparticle systems. In particular, plant-derived exosomes have attracted increasing interest relative to animal-derived exosomes due to their lower production costs, favorable safety profile, and generally non-toxic and non-immunogenic nature (Cai et al., 2022). In addition, plant-derived exosomes are not known to cross the placental barrier, further supporting their potential safety for clinical application (Cai et al., 2022).

Several studies have demonstrated the therapeutic potential of exosome-based delivery systems in experimental models of IBD. For example, exosomes derived from human bone marrow–derived mesenchymal stromal cells were shown to attenuate inflammatory responses and preserve intestinal barrier integrity in murine models of colitis (Liu et al., 2019). In another study, mesenchymal stem cell–derived exosomes loaded with miR-27a-3p maintained epithelial viability and barrier function in patient-derived colonoid cultures, without inducing cytotoxicity or structural disruption (Oh et al., 2025). Collectively, these findings highlight the promising application of exosome-based nanoparticles as safe and effective drug delivery vehicles for IBD therapy.

### 3.1 Limitations of Nanotechnology

Despite the growing interest in nanotechnology-based approaches for the diagnosis and treatment IBD, substantial biological, technical, and translational barriers continue to hinder their successful clinical adoption.

### 3.2 Gastrointestinal physiological barriers

The GI tract presents a highly dynamic and heterogeneous environment, particularly under inflammatory conditions. Inflammation-associated changes such as altered epithelial permeability, mucosal ulceration, bleeding, and edema, together with marked heterogeneity in histopathological features—including mucus thickness, enzymatic activity, oxidative stress levels, and microbiota composition—complicate the predictable behavior of NPs across different disease stages and phenotypes (Barros et al., 2026). In addition, rapid luminal transit, clearance, and dilution effects can substantially reduce NP residence time at inflamed sites, limiting local drug accumulation and therapeutic efficacy.

### 3.3 Off-Target effects and limited targeting specificity

Many NP-based delivery systems rely on passive targeting mechanisms analogous, which are highly variable in the inflamed gut and lack spatial precision (Lin et al., 2025). Although active targeting strategies employing antibodies, peptides, or other ligands have been explored, these approaches are often compromised by ligand instability or loss of functionality within the harsh GI environment. Furthermore, nonspecific uptake by immune cells such as macrophages in non-inflamed or off-target tissues, can diminish therapeutic selectivity and increase systemic exposure and adverse effects.

### 3.4 Limitations of stimuli-responsive nanoparticle designs

Stimuli-responsive NP systems, including those triggered by pH, enzymatic activity, or ROS, depend on pathological thresholds that vary widely among patients, disease locations, and inflammatory states (Barros et al., 2026). Significant overlap between physiological conditions in healthy and inflamed tissues may result in premature, delayed, or incomplete drug release. Consequently, achieving robust and reproducible site-specific activation remains a major challenge for these smart delivery platforms.

### 3.5 Insufficient diagnostic sensitivity and clinical validation

NP-based diagnostic and imaging platforms often suffer from limited sensitivity in the inflamed intestine due to high background inflammation, tissue autofluorescence, and nonspecific signal interference. Moreover, correlations between NP-derived diagnostic readouts and established clinical endpoints—such as endoscopic severity, histological inflammation, or treatment response—remain insufficiently validated. As a result, most NP-based diagnostic systems are confined to preclinical studies, with limited translation into human clinical trials.

### 3.6 Safety and toxicity concerns

Given the chronic and relapsing nature of IBD, long-term or repeated administration of NP-based formulations raises persistent safety concerns. Potential nanotoxicity includes tissue accumulation, induction of oxidative stress, inflammatory responses, genotoxicity, and cytotoxicity. Importantly, NP-associated toxicity is highly dependent on particle composition, size, surface chemistry, and biodegradability (Cai et al., 2022; Reindl et al., 2025). For instance, iron oxide nanoparticles and exosome-derived nanoparticles have generally demonstrated minimal cytotoxicity in biological systems, underscoring the importance of rational NP design in mitigating adverse effects. Nevertheless, standardized and disease-relevant toxicity assessment frameworks for orally administered nanomedicines are still lacking.

### 3.7 Cost, scalability, and accessibility

Advanced NP platforms frequently involve expensive materials, sophisticated fabrication techniques, and multistep processing, which limit scalability and increase production costs (Belal and Gad, 2023; He et al., 2019; Huang et al., 2024). Consequently, the cost-effectiveness of NP-based therapies relative to established biologics and small-molecule treatments remains largely unproven, raising concerns about real-world accessibility and health system adoption. Furthermore, different classes of nanoparticles present distinct challenges in terms of scalability and cost. Polymeric nanoparticles often rely on multistep synthesis and solvent-based fabrication processes, which complicate large-scale reproducibility and efficient solvent removal. In contrast, lipid-based nanoparticles require precise control over composition and particle size during rapid mixing, a requirement that is difficult to maintain consistently at industrial scale. Inorganic metal nanoparticles pose additional challenges related to batch-to-batch variability, residual impurities, and stringent safety and quality control requirements. Biologically derived nanoparticles, such as exosome-based systems, present even greater scalability barriers due to limited source material, complex and low-yield isolation procedures, and high production costs.

### 3.8 Manufacturing and regulatory challenges

Many NP formulations require complex synthesis and characterization processes that are difficult to reproduce consistently under Good Manufacturing Practice (GMP) conditions (Ball et al., 2017; Cui et al., 2022; Li et al., 2022). Batch-to-batch variability in particle size, surface properties, and drug loading can significantly impact therapeutic performance and reproducibility. Regulatory oversight of nanomedicines is primarily conducted by the U.S. Food and Drug Administration (FDA) and the European Medicines Agency (EMA), with a focus on quality, safety, biocompatibility, biodegradation, and environmental toxicity. In addition to standard investigational medicinal product requirements, NP-based formulations entering clinical development must provide detailed manufacturing parameters and comprehensive physicochemical characterization, as *in vitro* quality attributes do not necessarily predict *in vivo* behavior (Csóka et al., 2021; Dri et al., 2023). However, most current regulatory guidance for nanomedicines is derived from conventional drug development frameworks and does not fully account for the physicochemical complexity of NP. The absence of standardized definitions and harmonized classification criteria often limited to size-based descriptors—remains a key challenge, as these fail to capture material properties with clinically relevant implications.

### 3.9 NP type diversity and public acceptance

A wide range of NP–based drug delivery systems have been investigated for IBD, each characterized by distinct physicochemical properties, therapeutic efficiencies, and safety profiles (**Table 2**) (Alshawwa et al., 2022). Variability in NP composition, size, surface chemistry, biodegradability, and payload capacity results in differing levels of drug delivery efficiency and toxicity across NP platforms. Consequently, broad generalizations regarding the efficacy or safety of NP-based therapies are inherently problematic. This heterogeneity complicates regulatory evaluation and contributes to public and patient skepticism, as concerns regarding long-term safety, environmental impact, and biological accumulation remain highly NP-specific. Addressing these challenges will require transparent risk–benefit communication, standardized safety assessment frameworks, and NP-type–specific clinical evidence to improve public trust and acceptance.

In summary, while nanomaterials hold significant promises for drug delivery, diagnostics, and targeted therapies—particularly for chronic GI conditions—critical challenges remain, including large-scale production, manufacturing consistency, and ensuring long-term product stability (Korsmeyer, 2016; Landesman-Milo and Peer, 2016). Continued research is essential to address these limitations and fully realize the potential of NP-based approaches (Huang et al., 2024).

## 4 A living diagnostic-therapeutic system

**Figure 4:**
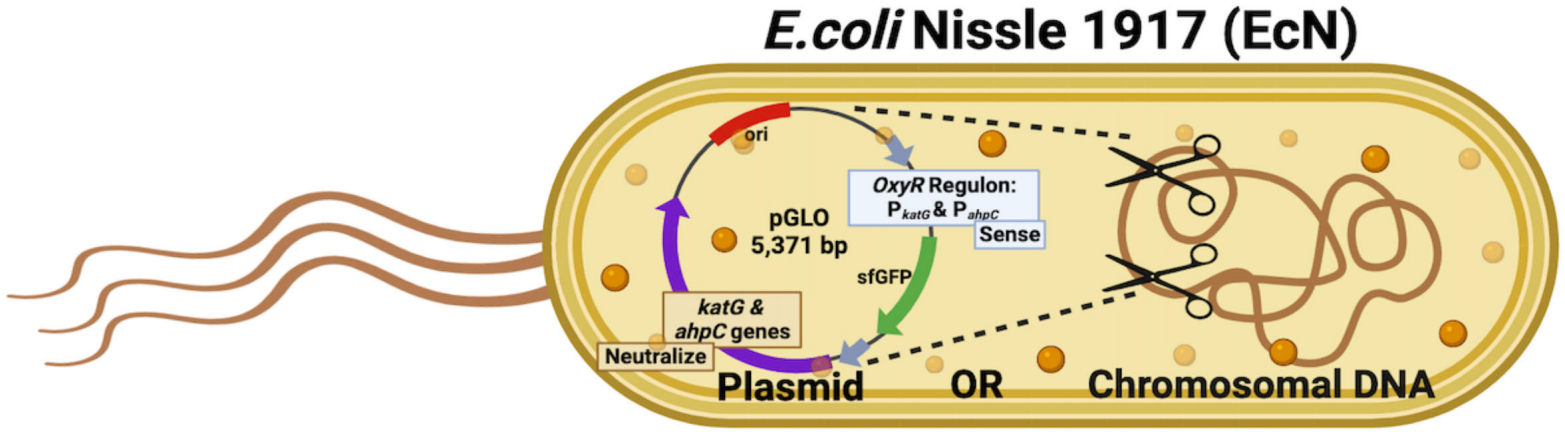
An optimal probiotic construct designed for the development of a living diagnostic–therapeutic platform. Both plasmid-based and genomic-insertion strategies have been utilized across different studies (Chen et al., 2023b; Petersen et al., 2014; Tursi et al., 2022; Petersen, 2023; Liang et al., 2022; Yang et al., 2022). The figure was created using BioRender.

A living diagnostic system is an emerging area of research in which genetically engineered bacterial species are used to detect biological or chemical reactions within the mammalian body by producing signals proportional to the concentration of a specific analyte (Bhalla et al., 2016). Following successful proof-of-concept studies in several indications, engineered living systems are now being expanded to not only continuously monitor biological environments but also respond dynamically when and where needed. In this context, beneficial bacterial species—often probiotics—are genetically modified to develop complex, real-time sensing and therapeutic systems (**Figure 4**). This approach leads to the concept of a DX-RX system (Diagnose and Prescribe), also referred to as a living diagnostic-therapeutic system. The advantages of such living systems include real-time monitoring and dynamic response, targeted therapy delivery, adaptive and personalized treatment strategies, improved disease management, and reduced healthcare burden (Almekkawy et al., 2020; Alvarez-Olmos and Oberhelman, 2001; Villalobos-Quesada et al., 2023; Zhou et al., 2020). Living diagnostic-therapeutic biosensors offer a powerful and minimally invasive approach for real-time, continuous monitoring, and early detection of disease conditions. These biosensors can be ingested, worn, or implanted (Nguyen et al., 2021), enabling personalized treatment with improved accuracy and a reduced need for external intervention. Functioning as internal surveillance systems, they detect physiological changes and can deliver therapy on demand, making them promise for managing inflammatory diseases and even cancer (Bhatia et al., 2024; Hemdan et al., 2024; Howarth et al., 2005; Senf et al., 2020). Engineered with well-defined gene-regulatory networks and synthetic feedback modules, these biosensors can offer a dynamic sensing and therapeutic responses, unlike natural probiotics, which produce constant response from poorly understood systems (Landry and Tabor, 2017).

There have been significant advancements in the field of synthetic biology in recent years, and the engineering of probiotics represents the next generation of development in live biotherapeutics (Aggarwal et al., 2020). Genetically engineered probiotics can be equipped with sensing features endowed by synthetic genetic circuits specifically engineered to detect a wide range of environmental cues originating from various pathological conditions (Debnath et al., 2024). These engineered probiotics also show potential for treating difficult-to-manage conditions, including metabolic disorders, IBD, multiple sclerosis, and a range of bacterial infections (Ma et al., 2022). These systems are designed to both detect and provide therapeutic response against the underlying health conditions, functioning as part of a living diagnostic-therapeutic (DX-RX) system. Programmable probiotics offer several advantages: they are internal, provide real-time responses, are self-replicating, site-specific, non-chemical, non-invasive, and easy to administer with minimal patient preparation. They are tunable for sensitivity and are biocompatible (Garg et al., 2021; Pesce et al., 2022). For example, some probiotic biosensors are engineered to detect ROS such as hydrogen peroxide (H_2_O_2_) (Semwal and Gupta, 2021).

Biosensors are generally categorized into two main types: Catalytic-based biosensors, which involve enzymes, microbes, organelles, or tissues; and Affinity-based biosensors, which rely on antibodies, receptors, or nucleic acid based aptamers (Hasan et al., 2014). For the purposes of this review, we focus solely on catalytic-based biosensors.

Engineered whole cell microbes have been applied as probiotics in several formats. They have been engineered to confer unique capability to inhibit pathogens by secreting antimicrobial peptides and bacteriocins, which provide them with a growth advantage over other commensal bacterial species (Fang et al., 2018; Winter et al., 2010; Ott et al., 2004). Probiotics have been also applied to support immune regulation and may help prevent the progression of inflammatory diseases (Suez et al., 2019; Tsai et al., 2019; Shamoon et al., 2019). Lactobacilli probiotics generate various acids such as lactic, acetic, and propionic acids, that lower pH and suppress the growth of various pathogens, particularly gram-negative bacteria (De Keersmaecker et al., 2006; Shahid et al., 2017). Probiotics can therapeutically modulate the gut microbiome by degrading environmental pollutants, neutralizing microbial toxins, and alleviating disease symptoms (Romero-Luna et al., 2022; Kumar et al., 2016; Kohl et al., 2020). Advances in synthetic biology have enabled the development of engineered probiotic strains with specialized functions, including disease treatment and tumor targeting, which was impossible before (Liu et al., 2023; Chung et al., 2021; Mazhar et al., 2020).

EcN is the only well-established Gram-negative probiotic bacterium, known for being easily manipulated, transformed, and cultured, with proven safety and robust colonization potential (Scaldaferri et al., 2016; Zhang et al., 2012; Secher et al., 2017; Danino et al., 2015). Hence, EcN is one of the most extensively studied and engineered probiotic strains. EcN is the primary component of the commercial probiotic product Mutaflor, which has been used to manage various gastrointestinal disorders, including IBD, diarrhea, uncomplicated diverticular disease, and UC. In some cases, it has also contributed to digestive disease remission (Jacobi and Malfertheiner, 2011; Dembiński et al., 2016; Park, 2021). The therapeutic potential of EcN is attributed to several key characteristics: anti-inflammatory activity, support for intestinal barrier function, immuno-modulatory effects, ability to colonize and regulate the human gut microbiota, production of antimicrobial compounds, and strengthening of the epithelial barrier (Chen et al., 2023b; Han et al., 2024; Helmy et al., 2017; Hu et al., 2020; Kandasamy et al., 2016, 2017; Kumar et al., 2023; Park et al., 2021; Pradhan and Alison, 2020; Sonnenborn and Schulze, 2009; Vlasova et al., 2016). Although efforts have been made to develop probiotic biosensors, the integration of dynamically controlled genetic circuits capable of recognizing biomarkers and producing therapeutic molecules locally is still in its early stages (Liu et al., 2023). Below, we describe some of the potential engineered probiotic studies along with their outcomes. **Tables 3** and **4** summarize the engineered probiotics used to treat specific GI disease conditions in both human and animal models. Given the prevalence of studies focusing on EcN, we have grouped all engineered EcN-based strains into one category, while other engineered probiotic species are presented in a separate group.

**Table 3:** Summary of human clinical trials utilizing engineered probiotics for the treatment or diagnosis of gut inflammatory conditions. The current outcomes of these trials are presented.

Species used	Detection: therapeutic or diagnostic	Condition	Outcome	Reference
*EcN*	Therapeutic	Phenylketonuria	N/A	(Zhou, 2023)
*EcN*	Therapeutic	Ulcerative Colitis on 5-ASA	Adding EcN didn’t improve quality of life, but helped prevent worsening, improved early symptoms and healing, and was safe.	(Park, 2021; Park et al., 2022b; Park, 2022a)
*EcN*	Therapeutic	Minimal Hepatic Encephalopathy	EcN is a safe, effective probiotic that may improve mild hepatic encephalopathy and serve as an alternative or add-on to standard treatments.	(Kobyliak, 2021; Manzhalii et al., 2000)
*EcN*	Therapeutic	Ulcerative Colitis	EcN showed no added benefit for active ulcerative colitis and led to lower remission rates without prior antibiotic treatment.	(Petersen, 2013, 2023; Petersen et al., 2014, 2009; Vejborg et al., 2011)
*EcN*	Therapeutic	Colonic Diverticulitis	N/A	(Spaziani, 2023; Kvasnovsky et al., 2015; MJ et al., 2022; Tursi et al., 2022; Teng et al., 2022; Lahat et al., 2020)
*EcN*	Therapeutic	IBD	N/A	(Garnås, 2016)
Other Species Used	Detection: Therapeutic or Diagnostic	Condition	Outcome	Reference
*L. reuteri*	Prevention	IBD	N/A	(Chen, 2025)
*L. acidophilus* and *L. rhamnosus*	Therapeutic	Crohn’s Disease	N/A	(Klinge, 2006b)
*L. reuteri*	Prevention	Ulcerative Colitis	N/A	(Hellström, 2019)
*L. acidophilus* and *L rhamnosus*	Therapeutic	Ulcerative Colitis	N/A	(Klinge, 2006a)
*Bifidobacterium longum* subspecies infantis	Prevention	Inflammatory Conditions	N/A	(Philipsen, 2024)
*Saccharomyces boulardii*	Therapeutic	Mild forms of Ulcerative Colitis and Crohn’s Disease	N/A	(Panic, 2019)
Lactobacilli	Therapeutic	Inflammatory Depression	Found no significant differences between probiotics and placebo on any of the primary or secondary outcomes.	(Lindqvist, 2023; Söderberg Veibäck et al., 2025)
*B. breve*	Therapeutic	Crohn’s Disease	After 8 weeks there was no effect on Crohn’s clinical outcomes or small intestine bowel wall thickness versus placebo.	(Petersen, 2023; Grønbæk et al., 2025)

**Table 4:** Summary of animal trials utilizing engineered probiotics for the treatment or diagnosis of gut inflammatory conditions. The current outcomes of these trials are presented.

Species used	Detection: therapeutic or diagnostic	Condition	Outcome	Experiment details	Reference
*EcN*	Therapeutic	Intestinal Inflammation	Relieved inflammation.	*In Vivo*: Mice	(Zhou et al., 2022)
*EcN*	Therapeutic	IBD	Effectively secreted IL-2, relieved IBD, showed therapeutic efficiency.	*In Vivo*: Mice	(Li et al., 2024)
*EcN*	Therapeutic	Colitis	Improvement of colonic microenvironments and further treatment of colitis was seen.	*In Vivo*: Mice	(Yan et al., 2021)
*EcN*	Therapeutic	IBD, Fistulae and Ulcers	EcN secreted the curli-fused TFFs *in vitro* and *in vivo*. Protective effects were seen in mice.	*In Vitro*: human colorectal adenocarcinoma & *In Vivo*: Mice	(Praveschotinunt et al., 2019)
*EcN*	Therapeutic/Diagnostic	Colorectal Neoplasia	Selective colonization, detection, and reduction of colorectal adenomas.	*In Vitro*: colorectal cancer organoids mouse fecal & human stool *In Vivo*: Mice	(Gurbatri et al., 2024)
*EcN*	Therapeutic	Ulcerative Colitis	Enhanced anti-inflammatory ability *in vivo* and improved the colonization rate.	*In Vitro*: U2OS & *In Vivo*: Mice	(Zhao et al., 2022)
*EcN*	Therapeutic	Colitis	Restored intestinal redox balance and immune regulation homeostasis.	*In Vitro*: HT29 & *In Vivo*: Mice	(Xu et al., 2023)
*EcN*	Therapeutic	IBD	Enhanced colonization and controlled CO/H_2_S release at inflamed sites. Mitigated anxiety-depression behaviors.	*In Vivo*: Mice	(Ma et al., 2025)
*EcN*	Therapeutic	IBDs	EcN coated with zinc & indole-3-carbinol improved colon retention, restored epithelial barrier, reduced colonic inflammation, and increased beneficial bacteria.	*In Vitro*: Caco-2, NCM460, and HCT116 & *In Vivo*: Mice	(Chen et al., 2025)
*EcN*	Therapeutic	Inflammation and Restoration of Gut Microbiota	Enhanced the colonic epithelial barrier, promoted resolution of inflammation, modulated the gut microbiota, and elevated concentrations of short-chain fatty acids.	*In Vitro*: Caco-2 & *In Vivo*: Mice	(Teng et al., 2022)
*EcN*	Therapeutic	IBD	Elevated indole metabolites, improved intestinal permeability, reduced inflammation, and recovered the gut microbiome.	*In Vitro*: NCM460 & *In Vivo*: Mice	(Li et al., 2024)
*EcN*	Therapeutic	Colitis	Sequestrated and neutralized multiple inflammatory cytokines. Alleviated IBD symptoms and enhanced gut microbiota recovery.	*In Vitro*: Caco-2 & *In Vivo*: Mice	(Gu et al., 2024)
*EcN*	Therapeutic	IBD	Inhibited neutrophil chemotaxis and ROS production.	*In Vitro*: Caco-2 & *In Vivo*: Zebrafish	(Chen et al., 2023a)
*EcN*	Therapeutic	Ulcerative Colitis	Enhanced therapeutic efficacy, downregulated proinflammatory cytokines, and repaired the intestinal barrier. Increased the diversity of beneficial microbes.	*In Vivo*: Mice	(Chen et al., 2023)
*EcN*	Therapeutic	Colitis	Enhanced intestine retention time of EcN, which improved bacterial diversity and shifted the microbiota community and metabolites to an anti-inflammatory phenotype.	*In Vitro*: Caco-2 & *In Vivo*: Rat	(Wang et al., 2023)
*EcN*	Therapeutic	Colitis	Significant attenuation of clinical activity of colitis, enhanced microbiota diversity and butyrate production.	*In Vitro*: Engineered EcN & *In Vivo*: Mice	(Wang et al., 2021)
*EcN*	Therapeutic	IBD	Demonstrated GI tract tolerance and robust bioactivity.	*In Vitro*: Engineered EcN & *In Vivo*: Mice	(Su et al., 2025)
*EcN*	Therapeutic	Colitis	Ameliorated intestinal inflammation and modulated the gut microbiome.	*In Vitro*: Caco-2 & *In Vivo*: Mice	(Yin et al., 2024)
*EcN*	Therapeutic/Diagnostic	Colitis	Alleviation of clinical symptoms and pathological abnormalities. Restored mucosal integrity and upregulated tight junctions.	*In Vivo*: Mice	(Liang et al., 2022)
*EcN*	Therapeutic	Colitis	Down regulation of inflammation and an increase in abundance and diversity of gut microflora.	*In Vitro*: Engineered EcN & *In Vivo*: Mice	(Yang et al., 2022)
*EcN*	Therapeutic	Hyperammonemia in CLD	Inflammation reduction.	*In Vivo*: Rat	(Ochoa-Sanchez et al., 2021)
*EcN*	Therapeutic	Restoration of intestinal barrier	Promoted recovery of damaged epithelial layers, barrier, and mucosal integrity.	*In Vitro*: HCT-8 & *In Vivo*: Mice	(Yu et al., 2019)
*EcN*	Therapeutic	IBD	Alleviation of intestinal inflammation and a restored epithelial barrier. Enhanced diversity of the intestinal flora, short-chain fatty acids.	*In Vitro*: Engineered EcN & *In Vivo*: Mice	(Liu et al., 2025)
*EcN*	Therapeutic	Colorectal Cancer	Showed weight loss, and reduction of tumorigenesis. Enrichment of beneficial bacterial population.	*In Vitro*: CT26 & *In Vivo*: Mice	(Tang et al., 2023)
*EcN*	Therapeutic/Diagnostic	Colorectal Cancer	Inhibition of tumor growth, an increased colon length and decreased polyp count.	*In Vitro*: CRC & *In Vivo*: Mice	(Zhou et al., 2024)
*EcN*	Therapeutic	Gastrointestinal Disorders	Constipation related symptoms relieved, and GI motility enhanced. Improved microbiota composition.	*In Vitro*: Engineered EcN & *In Vivo*: Mice	(Li et al., 2022)
Other Species Used	Detection: Therapeutic or Diagnostic	Condition	Outcome	Experiment Details	Reference
*S. cerevisiae*	Therapeutic	IBD	Suppressed intestinal inflammation, reduced intestinal fibrosis, and dysbiosis.	*In Vivo*: Mice	(Scott et al., 2021)
*B. longum*	Therapeutic/Diagnostic	Intestinal Inflammation and Microbiota Dysbiosis	Reshaped the intestinal barrier functions and restored gut microbiota.	*In Vitro*: HT29 & *In Vivo*: Mice	(Cao et al., 2023)
*S. cerevisiae*	Therapeutic	Ulcerative Colitis	Showed anti-inflammatory, improved histological lesions, increased mucosal barrier, and decreased intestinal immune response.	*In Vivo*: Mice	(Sun et al., 2021)
*L. plantarum*	Therapeutic	Ulcerative Colitis	Ameliorated UC, improved mucosal barrier and inflammation.	*In Vivo*: Mice	(Wu et al., 2022)
*L. plantarum*	Therapeutic	Ulcerative Colitis	Alleviated colitis symptoms, reduced histological lesions, and improved microbiota diversity.	*In Vivo*: Mice	(Hao et al., 2021)
*S. boulardii*	Therapeutic	Colitis	Improvement in colon length, and histological inflammation scores	*In Vitro*: HT-29 *In Vivo*: Mice	(Heavey et al., 2024)
*Akkermansia muciniphila*	Therapeutic	Colitis	Improved colitis symptoms, colonic tissue injury and intestinal barrier effectiveness. Beneficial bacteria increased.	*In Vitro*: RAW264.7 & *In Vivo*: Mice	(Zheng et al., 2023)
*L. plantarum*	Therapeutic	Colitis	Protection against inflammatory injury and goblet cell loss, with an activation of the inflammasome during colitis.	*In Vitro*: RBL-2H3 *In Vivo*: Mice	(Yin et al., 2024)
*S. cerevisiae*	Therapeutic	IBD	Regulated gut microbiota and butyrate content. Increased abundance of probiotic bacteria.	*In Vitro*: BYJ10 *In Vivo*: Mice	(Wu et al., 2024)
*S. boulardii*	Therapeutic	Colitis	Amelioration of the colitis. improved body weight, disease activity index, and survival rate.	*In Vivo*: Mice	(Liu et al., 2020)
*Lactobacillus rhamnosus*	Therapeutic	Colon Cancer	Improvement of survival, reduced tumor progression, and alleviated muscle wasting was seen. Gut microbial diversity, increased abundance of beneficial bacteria, and production of short-chain fatty acids.	*In Vivo*: Mice	(Qiu et al., 2025)
*S. cerevisiae* var. *boulardii*	Therapeutic	Colitis	Superior in maintaining a stable gut microbial diversity upon inflammation.	*In Vivo*: Mice	(Deleu et al., 2024)
*S. boulardii*	Therapeutic	Intestinal Inflammation	Low disease activity index score, prevention of colon shortening, and reduction in pro-inflammatory cytokines.	*In Vivo*: Mice	(Han et al., 2025)

In one recent study, researchers engineered and characterized a synthetic genetic circuit in the EcN. This engineered strain was able to detect nitric oxide (NO) as a biomarker and respond by producing and secreting nanobodies that sequester TNFα, thereby reducing inflammation at the local site (Weibel et al., 2024). These findings highlight the potential of engineered probiotic as a targeted, real-time and a living biotherapeutic.

To manage IBD, researchers developed a fluorescent reporter system using *E. coli* by engineering fimbriae and NO-responsive regulator NorR to respond to NO an inflammatory signal. This engineered system successfully detected NO in the gut, indicating inflammation (Archer et al., 2012). A similar approach was used to detect tetrathionate, a byproduct of ROS generated during gut inflammation. In this study, researchers engineered a mouse-derived *E. coli* strain capable of sensing tetrathionate in the inflamed gut of mice (Riglar et al., 2017). In a separate study, EcN was engineered to overexpress catalase and superoxide dismutase for the treatment of inflammatory conditions in the gut. To enhance its stability and bioavailability in the GI tract, the engineered EcN was coated with chitosan and sodium alginate (Zhou et al., 2022). The obtained results demonstrate that this engineered coated EcN alleviated acute IBD in mice and significantly increased the abundance and richness of beneficial gut bacteria. This study provided foundational evidence that orally delivered, biofilm-coated engineered probiotics can regulate gut inflammation and relieve acute colitis in a mouse model (Zhou et al., 2022).

These studies highlight the potential of engineered bacteria as a therapeutic tool to treat and manage intestinal cancers, with the hope of future clinical translation in humans. Besides, in the above applications, engineered probiotics also have been utilized to bypass pathogen defenses through mechanisms such as anti-quorum sensing, anti-biofilm activity, antibiotic production, quorum sensing modulation, and signal interference (Salman et al., 2023; Vestby et al., 2020; Gao et al., 2019; Rizzello et al., 2014).

### 4.1 Challenges in living biosensor systems

Genetically engineering microbes or probiotics is an emerging and promising field, particularly in the early stages of development. However, significant challenges remain before biosensor technologies can be successfully translated into clinical medicine. Key challenges include sensitivity and detection limits, signal stability, detection time, multiplexing capability, continuous *in vivo* monitoring, environmental sustainability, biocompatibility in living organisms, cost-effectiveness, and integration with devices and commercialization (Liu, 2021; Varshney and Mallikarjunan, 2009; Chen et al., 2024). To be clinically viable, biosensors must be highly specific to the target site and activate only when required. Additional hurdles include response time (Wu et al., 2019), sample preparation, and sample matrix effects particularly in microfluidic chip-based systems as well as ensuring minimal side effects (Sin et al., 2014). Maintaining minimal side effects, while achieving accurate long-term monitoring, remains a critical concern.

### 4.2 Functionality and genetic stability

A 2024 study (Weibel et al., 2024) highlights several intrinsic biological challenges: consistent therapeutic delivery, stable colonization in the gut, metabolic burden on the engineered host, and the risk of evolutionary changes or genetic mutations in the host that reduce treatment efficacy (Ceroni et al., 2015; Sleight and Sauro, 2013; Sleight et al., 2010). One proposed solution is to insert synthetic circuits into the host genome, which may improve stability and robustness of function (Triassi et al., 2023; Ingram and Stan, 2023). However, challenges still persist, particularly in maintaining long-term expression, preventing loss of function, and minimizing genetic drift (Riglar et al., 2017). Designing mutation-resistant synthetic circuits may improve genetic stability, especially under cellular stress conditions (Brooks and Alper, 2021). Research is also focused on improving stability through protective coatings that resist degradation engineered strain in the GI tract (Bhatia et al., 2024).

### 4.3 Colonization and host interaction

Therapeutic implementation also faces difficulties with reliable colonization, dosage control, strain-to-strain variation, and effective host interaction (Suez et al., 2019). Improving long-term colonization in the human gut would greatly enhance the clinical applicability of engineered probiotics (Mimee et al., 2016). On the other hand, selecting a probiotic strain with better colonization ability could address these concerns, as studies have shown differences in probiotics’ ability to colonize and induce beneficial effects (Kandasamy et al., 2016, 2017).

### 4.4 Manufacturing and healthcare integration constraints

From an economic standpoint, biosensor development also involves high production costs (Thenrajan and Wilson, 2022) and complex sensor fabrication. Integrating biosensors into current healthcare frameworks, especially those governed by FDA regulations, adds another layer of complexity. Key concerns include system compatibility, data standardization, communication between biosensors and clinical data systems. Such integration is essential for real-time decision-making, improved diagnostics, and patient care outcomes (Bhatia et al., 2024).

### 4.5 Regulatory and translational challenges

Translation of genetically modified living diagnostic–therapeutic systems into clinical practice faces significant regulatory hurdles. Oversight by agencies such as the FDA, EMA, and the World Health Organization (WHO), along with country-specific regulatory bodies, complicates clinical approval and commercialization. Furthermore, public and consumer perceptions of living, genetically modified organisms differ across regions and may impact regulatory decision-making and clinical implementation (Bawa and Anilakumar, 2013; Marette et al., 2021). These systems are regulated differently from conventional small-molecule drugs and biologics due to concerns related to biosafety, genetic stability, long-term host interactions, and environmental containment (Cruz et al., 2022).

Unlike traditional therapeutics, evaluation of living systems requires specialized consideration of dosing, pharmacodynamics, and pharmacokinetics, including absorption, distribution, metabolism, and excretion. In addition, therapeutic performance is strongly influenced by host-specific factors such as microbiome composition, diet, immune status, and lifestyle, resulting in substantial inter-individual variability. Addressing this variability will likely require a shift from population-based treatment paradigms toward personalized medicine approaches, including patient stratification based on microbiome and physiological profiles. Although these technologies show considerable promise, they remain at an early stage of clinical translation. Broader adoption will depend on the evolution of regulatory frameworks, improved biocontainment strategies, and deeper understanding of host–microbe interactions.

In summary, while genetically engineered probiotics and living biosensors represent a transformative direction in precision medicine, particularly for chronic disease like IBD, their clinical translation remains hindered by several biological, technical, regulatory, and economic challenges. Continued innovation in synthetic biology, circuit stability, host compatibility, and sensor integration will be essential for overcoming these barriers.

## 5 Conclusion

Chronic inflammatory disorders of the gastrointestinal tract are increasing globally, driven in part by changes in lifestyle, dietary patterns, and environmental exposures, resulting in a rapidly evolving disease epidemiology. Current diagnostic approaches remain limited in their ability to provide real-time, longitudinal assessment of disease activity, while existing therapeutic strategies largely rely on long-term systemic administration of anti-inflammatory or immunosuppressive agents, often accompanied by significant adverse effects. Emerging technologies, including NP-based diagnostic and therapeutic platforms, offer promising opportunities to address several limitations of conventional approaches by enabling targeted delivery and enhanced disease-site specificity. In parallel, living diagnostic–therapeutic systems based on whole-cell sensors represent an innovative strategy for real-time detection and on-demand intervention in chronic inflammatory conditions, although still in their infancy, early preclinical and limited clinical studies have demonstrated encouraging potential. Nevertheless, both NP-based and living therapeutic approaches face substantial challenges related to safety, scalability, regulatory approval, and clinical translation. With growing interdisciplinary interest and continued efforts to address these barriers, the coming decade of research is likely to play a critical role in shaping the successful integration of these emerging technologies into real-world clinical practice.
